# Dual-block HER2 assessment reveals clinically relevant intratumoral heterogeneity in gynecologic cancers: a single-center landscape analysis

**DOI:** 10.1186/s12967-026-08546-8

**Published:** 2026-07-03

**Authors:** Xiaonan Zhou, Jing Gao, Ling Cui, Hui Li, Huijuan Ge, Lin Yu, Jiaojie Lv, Ke Zuo, Tian Tian, Yue Wang, Hui Sun, Yufan Cheng, Rui Bi

**Affiliations:** 1 Department of Pathology, Fudan University Shanghai Cancer Center, 270 Dong’an Road, Shanghai, 200032 China; 2 Shanghai General Hospital, Shanghai Jiao Tong University School, No. 100, Hai Ning Road, Shanghai, 200080 China

**Keywords:** HER2 heterogeneity, Immunohistochemistry (IHC), Gynecologic malignancies, Antibody drug conjugate

## Abstract

**Background:**

Gynecologic cancers remain a substantial clinical challenge, particularly in advanced stages, where treatment options are often associated with limited efficacy and poor prognosis. Given the emerging success of HER2-targeted antibody–drug conjugates (ADCs) across solid tumors, accurate evaluation of HER2 status is essential. This study investigated intratumoral HER2 heterogeneity in gynecologic cancers to refine detection accuracy and improve patient stratification for targeted therapy.

**Methods:**

A retrospective cohort of 416 patients with gynecologic malignancies was analyzed using immunohistochemistry (IHC) testing on separate dual formalin-fixed paraffin-embedded (FFPE) tumor blocks. HER2 expression was scored according to ASCO/CAP gastric criteria, and heterogeneity was defined as discordant IHC scores across blocks. Statistical analyses were performed using McNemar’s test, and clinical predictors of discordance were identified through multivariate logistic regression.

**Results:**

Across all tumors, HER2 IHC scores were distributed as 0 (49.5%), 1+ (33.9%), 2+ (15.6%), and 3+ (1.0%). HER2 overexpression was most frequent in uterine serous carcinoma, uterine endometrioid carcinoma, and ovarian clear cell carcinoma in our cohort. Intratumoral discordance was observed in 20.7% of cases, with the highest rates in uterine (23.9%, 21/88), ovarian (21.4%, 39/182), and cervical (18.2%, 26/143) tumors. Dual-block assessment revealed that most discrepancies resulted from incremental shifts in HER2 expression, primarily from 0 to 1 + or 1 + to 2+. This approach reclassified 13.5% of tumors originally reported as HER2-0 to HER2 expression.

**Conclusion:**

Intratumoral HER2 heterogeneity is common in gynecologic cancers and frequently results in underestimation of HER2 expression when single-block assessment is used. Dual-block evaluation improves detection sensitivity and may refine patient selection for HER2-targeted antibody–drug conjugates.

**Supplementary Information:**

The online version contains supplementary material available at https://doi.org/10.1186/s12967-026-08546-8.

## Highlights

A large retrospective cohort study of 416 surgically resected gynecologic tumors systematically evaluated HER2 intratumoral heterogeneity using dual-block immunohistochemistry (IHC).

Dual-block assessment revealed a 20.7% overall discordance in HER2 expression, with the highest variability in uterine (23.9%, 21/88), ovarian (21.4%, 39/182), and cervical (18.2%, 26/143) tumors.

Multi-block HER2 evaluation may improve the detection of HER2 expression by better capturing intratumoral heterogeneity and may provide a complementary approach for biomarker assessment in future prospective studies. However, its clinical implementation and utility for patient selection for HER2-targeted antibody–drug conjugate (ADC) therapy require further prospective validation.

**Supplementary Information:**

The online version contains supplementary material available at https://doi.org/10.1186/s12967-026-08546-8.

## Introduction

Gynecologic cancers—including endometrial, cervical, ovarian, and vulvar cancers—pose a major global health burden and are leading causes of cancer-related death in women, especially in advanced stages or recurrence where five-year survival remains below 30% [1]. Despite the developments in surgery, radiotherapy, and platinum-based chemotherapy, treatment options for patients with refractory or metastatic disease remain limited [2].

Recent advances in precision oncology have underscored the therapeutic potential of human epidermal growth factor receptor 2 (HER2) in cancers [3]. The DESTINY-PanTumor02 (DP-02) Phase II trial reported encouraging efficacy of T-DXd in HER2-expressing gynecologic tumors, with objective response rates (ORRs) of 84.6% (endometrial), 75.0% (cervical), and 63.6% (ovarian) [4]. Notably, patients with HER2 low expression also responded well, suggesting HER2 functions as a continuum rather than a binary biomarker [5].

However, intratumoral HER2 heterogeneity in solid tumors affects treatment response and resistance [6, 7]. Heterogeneous HER2 expression can compromise the accuracy of HER2 status assessment. Although HER2 spatial variability is well-studied in breast and gastric cancers, it is underexplored in gynecologic tumors. One study showed 13% discordance between FDA and ASCO/CAP IHC scoring, with concordance to FISH of 75% and 81%, respectively [8]. Another reported 24% discordance between NGS and IHC-FISH, attributed to intratumoral heterogeneity [9]. Immunohistochemistry serves as the most commonly used method for evaluating HER2 expression in clinical diagnosis. Emerging evidence suggests that dual/multi-block IHC testing may enhance the detection sensitivity and identify more HER2-targeted therapy candidates [10].

This study investigated the intratumoral HER2 heterogeneity in gynecologic cancers by performing IHC on at least two formalin-fixed paraffin-embedded (FFPE) tumor blocks per patient. Comparing HER2 expression across blocks, we sought to identify candidates for HER2-targeted therapies (e.g., T-DXd). These findings could inform the optimization of testing strategies and guide personalized treatment in gynecologic malignancies.

## Materials and methods

### Patient samples

A total of 416 patients who underwent surgical resection and were pathologically diagnosed with gynecologic cancers at our cancer center between July 2024 and July 2025 were retrospectively reviewed and included. The sample size was determined to ensure adequate precision for estimating intratumoral HER2 discordance. Based on previously reported HER2 heterogeneity across multiple regions or sections in other solid tumors (ranging from 15% to 30%), a conservative discordance rate of 20% was assumed [11]. Under this assumption, a minimum of 384 cases would be required to achieve a 95% confidence interval with a margin of error of ± 4%. The final cohort of 416 patients thus provided sufficient statistical robustness for the primary analyses. Tumor types included uterine, cervical, ovarian, and vulvar cancers. Patients were eligible for inclusion if they performed HER2 IHC tests on different paraffin blocks from the same tumor.

### Specimen processing and histological evaluation

Tumor tissue sampling followed standard pathological sampling principles for gynecologic specimens. During gross examination, tumor areas with obvious hemorrhage, extensive necrosis, or severe degenerative changes were excluded whenever possible to avoid compromised tissue quality. Multiregional tissue sampling was routinely performed, and most cases yielded 4–8 formalin-fixed paraffin-embedded (FFPE) tumor blocks to maximize spatial representation of the tumor. Before HER2 immunohistochemistry (IHC), all FFPE blocks were histologically reviewed on hematoxylin and eosin (H&E)-stained slides by experienced pathologists. Blocks were considered eligible for HER2 assessment if they contained abundant viable tumor tissue with preserved histomorphology and without extensive necrosis, hemorrhage, or marked degenerative changes that could compromise staining interpretation. To minimize potential selection bias, two tumor-containing blocks meeting these criteria were randomly selected from the eligible FFPE pool for HER2 IHC evaluation. No predefined minimal tumor size threshold was applied, provided that sufficient viable tumor cellularity was available for reliable pathological assessment.

Surgical specimens were fixed in 10% neutral-buffered formalin within 30 min of excision and processed using standard protocols following overnight fixation. H&E staining was performed according to institutional protocols. HER2 immunohistochemistry was performed using the rabbit monoclonal anti-HER2 antibody (clone 4B5, Roche Diagnostics, dilution 1:1200) on a Ventana Benchmark XT automated staining system (Ventana Medical Systems, Inc., Tucson, AZ, USA). Antigen retrieval was conducted using Cell Conditioning 1 (CC1; Tris-EDTA buffer, pH 8.5) according to the manufacturer’s standard automated protocol. All immunohistochemical runs included external HER2-positive (3+) control tissues and internal negative controls to ensure assay validity and reproducibility.

H&E-stained and HER2-immunostained slides were independently reviewed by two experienced pathologists who were blinded to clinical data. Discrepant cases were reviewed in a consensus session, and when disagreement persisted, a third senior pathologist performed adjudication.

### HER2 evaluation and heterogeneity assessment

Considering the therapeutic benefit of T-Dxd in gynecologic cancers demonstrated in the DP-02 study [4, 12], we assessed HER2 expression (scored as 0, 1+, 2+, or 3+) using the ASCO/CAP gastric cancer criteria-consistent with the approach employed in DP-02-rather than the specific diagnostic criteria for gynecological cancers [8]. HER2 expression was evaluated according to a standardized scoring system as follows [12]: 0, No reactivity or membranous reactivity in < 10% of tumor cells; 1+, Faint/barely perceptible membranous reactivity in ≥ 10% of tumor cells; cells are reactive only in part of their membrane; 2+, Weak to moderate complete, basolateral or lateral membranous reactivity in ≥ 10% of tumor cells; and 3+, Strong complete, basolateral or lateral membranous reactivity in ≥ 10% of tumor cells. In cases with discordant HER2 scores between the two blocks, the higher score was assigned as the final HER2 status to better capture intratumoral spatial heterogeneity and minimize the risk of underestimating HER2 expression. The estimated increase in detectable HER2 expression associated with dual-block assessment was calculated at the population level based on the reduction in HER2-0 classification rates relative to single-block evaluation.

A third pathologist re-evaluated all slides to confirm scores on the discordance HER2 scores. HER2 expression heterogeneity was defined as discordant IHC scores between the two blocks.

### Statistical analysis

To evaluate interobserver reproducibility of HER2 IHC scoring, slides were independently reviewed by two experienced pathologists who were blinded to clinical information. Interobserver agreement for the ordinal HER2 IHC scores (0, 1+, 2+, and 3+) was evaluated using the linearly weighted Cohen’s kappa statistic to account for the magnitude of disagreement between categories, with 95% confidence intervals (95% CIs) reported to reflect estimate precision. To compare the detection rates of different HER2 expression levels (0, 1+, 2+, and 3+) between single-block assessments (Block1 or Block2) and the final dual-block evaluation, the McNemar’s test was performed. For categories with small cell frequencies where the total number of discordant pairs was less than 25, McNemar’s exact test was applied to ensure statistical robustness. The 95% confidence intervals (95% CIs) for the proportional differences was calculated to quantify the effect sizes. To account for the multiple testing issue arising from parallel comparisons across four HER2 strata, P-values were further adjusted using the Benjamini-Hochberg (BH) method to control the False Discovery Rate (FDR). A two-tailed p-value < 0.05 was considered statistically significant. The effects of patient age and tumor type on discordance were further examined using multivariate logistic regression, with discordance (yes/no) as the dependent variable and age (continuous) and tumor type (categorical) as covariates. Owing to the extremely small sample size of vulvar cancer (*n* = 3), these cases were excluded from the regression analysis to avoid model instability and unreliable parameter estimation. Odds ratios (ORs) and 95% confidence intervals (CIs) were reported. Sankey diagrams were generated using the R packages ggplot2 and ggalluvial. Statistical analyses were conducted using SPSS version 25.0 and R version 4.2.2.

## Results

We evaluated HER2 expression by IHC in 416 gynecologic tumors. At least two FFPE blocks were analyzed for each case. The cohort included 88 uterine tumors (80 endometrioid carcinomas, 6 serous carcinomas, 1 clear cell carcinoma, 1 carcinosarcoma), 143 cervical tumors (121 squamous cell carcinomas, 19 adenocarcinomas and 3 adenosquamous carcinomas), 182 ovarian tumors (162 high-grade serous carcinomas, 2 low-grade serous carcinomas, 8 endometrioid carcinomas, 9 clear cell carcinomas and 1 mesonephric-like carcinoma), and 3 vulvar squamous cell carcinomas.

**Fig. 1:**
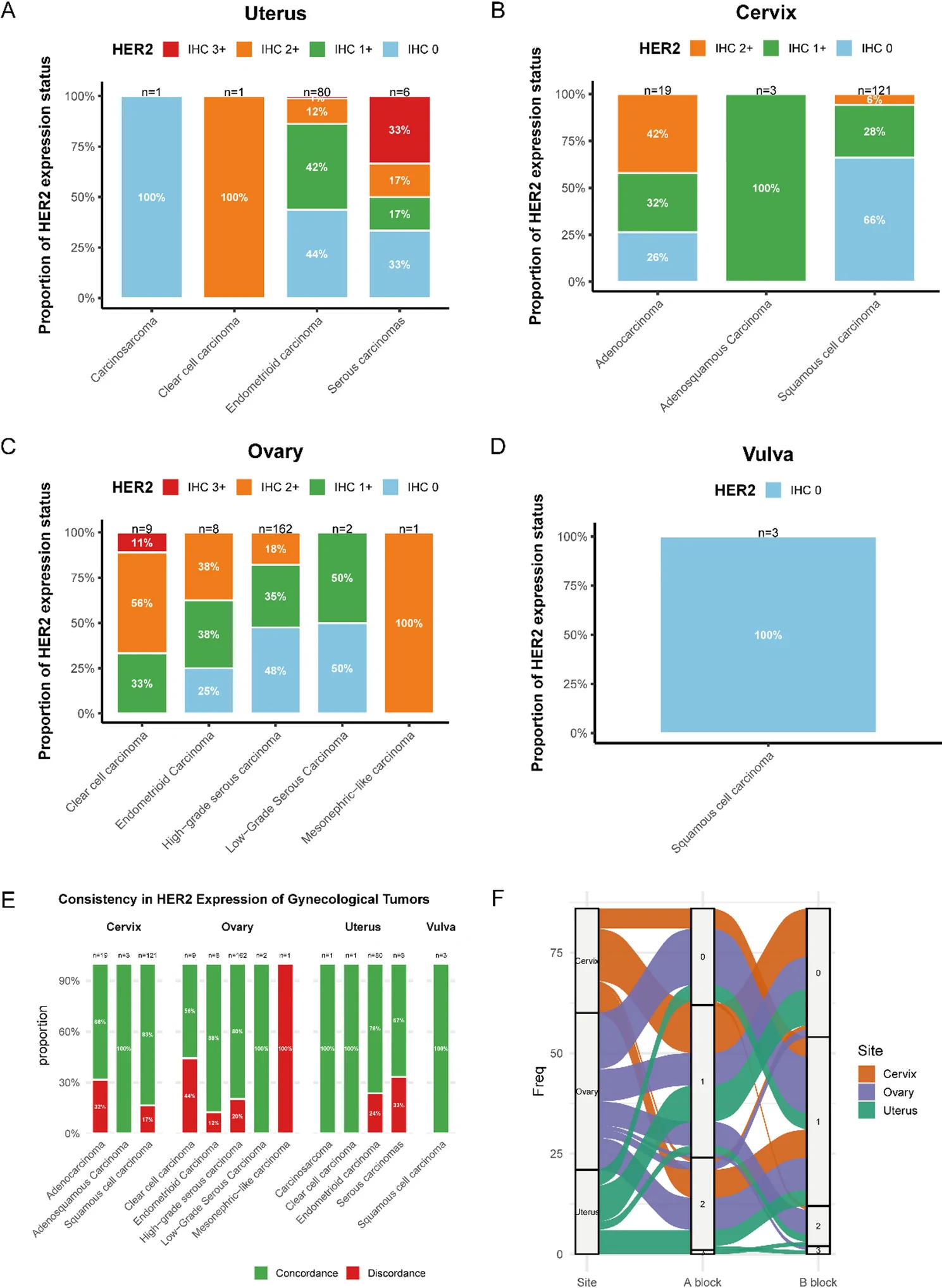
HER2 expression profiles across dual paraffin blocks in 416 gynecologic cancers. Distribution of HER2 expression status in different pathological types of gynecological tumors in (**A**) Uterus, (**B**) Cervix, (**C**) Ovary, (**D**) Vulva. (**E**) Concordance of HER2 IHC scores between dual/multiple tumor blocks. (**F**) Sankey diagram illustrating the pattern and distribution of HER2 discordance among the different gynecological tumors

Across all specimens, the distribution of HER2 IHC scores was as follows: 0 in 206 cases (49.5%), 1 + in 141 (33.9%), 2 + in 65 (15.6%), and 3 + in 4 (1.0%). Interobserver agreement on the ordinal HER2 scoring between the two reviewers was excellent (linear-weighted κ = 0.897, 95% CI: 0.869–0.922), confirming the high consistency and robustness of the IHC assessments throughout the study (Supplementary Table 1). Gynecologic malignancies represent tumor types with relatively low HER2 positivity rates. Among the 416 cases analyzed in our cohort, only four (1.0%) exhibited HER2 3+, including two uterine serous carcinomas, one endometrioid adenocarcinoma, and one ovarian clear cell carcinoma. In uterine carcinoma, HER2 3 + was most frequently observed in serous carcinoma, 2 + in clear cell carcinoma, 1 + in endometrioid adenocarcinoma, and 0 in carcinosarcoma. In cervical carcinoma, HER2 2 + was most common in adenocarcinoma, 1 + in adenosquamous carcinoma and 0 in squamous cell carcinoma. In ovarian carcinomas, HER2 3 + was mainly detected in clear cell carcinoma, and HER2 2 + was most commonly observed in Mesonephric-like carcinoma, while low-grade serous carcinomas predominantly exhibited HER2 1 + or 0. In the three cases of vulvar carcinoma, HER2 expression was consistently scored as 0 across all evaluated tissue blocks (Fig. 1A–D), indicating a uniformly negative staining pattern in this tumor subtype (Supplementary Table 2), which is further detailed in the overall distribution of HER2 expression across tumor types.

We further evaluated the concordance of intratumoral HER2 expression across all included cases (Table 1). Overall, HER2 IHC concordance between dual/multiple tumor blocks was 79.3% (330/416), with 20.7% (86/416) discordant. Discordance was highest among uterine cancers 23.9% (21/88), followed by ovarian tumors at 21.4% (39/182) and cervical tumors at 18.2% (26/143) (Fig. 1A). Within uterine cancers, serous carcinoma showed a slightly higher discordance rate (33.3%, 2/6) than endometrioid carcinoma (23.7%, 19/80). Among cervical tumors, adenocarcinomas demonstrated a discordance rate of 31.6% (6/19), exceeding that of squamous cell carcinomas (16.5%, 20/121), whereas all adenosquamous carcinomas (*n* = 3) were concordance. Among ovarian tumors, HER2 discordance was highest in mesonephric-like carcinoma (100%, 1/1), followed by clear cell carcinoma (44.4%, 4/9), high-grade serous carcinoma (20.4%, 33/162) and endometrioid carcinoma (12.5%, 1/8). All 3 vulvar squamous cell carcinomas showed complete concordance (Fig. 1E). After adjusting for age, tumor type was not significantly associated with HER2 IHC discordance (all *P* > 0.2), suggesting that inter-block heterogeneity was not driven by patient age or tumor histology but likely reflects intrinsic tumor or sampling variability (Supplementary Table 3). Among tumors with concordant HER2 expression, the majority exhibited 0 or 1 + staining. HER2 3 + positivity was observed in three uterine tumors and one ovarian tumor (Fig. 1C).

**Table 1:** HER2 expression in dual paraffin blocks in different histotypes in 416 cases

		Concordance (%)		Discordance (%)	Overall
					416
Uterus					88
	Endometrioid Carcinoma	61 (76.3)	19 (23.7)		80
	Serous carcinomas	4 (66.7)	2 (33.3)		6
	Clear cell carcinoma	1 (100)	0 (0)		1
	Carcinosarcoma	1 (100)	0 (0)		1
Cervix					143
	Squamous Cell Carcinoma	101 (83.5)	20 (16.5)		121
	Adenocarcinoma	13 (68.4)	6 (31.6)		19
	Adenosquamous Carcinoma	3 (100)	0 (0)		3
Ovary					182
	High-Grade Serous Carcinoma	129 (79.6)	33 (20.4)		162
	Low-Grade Serous Carcinoma	2 (100)	0 (0)		2
	Endometrioid Carcinoma	7 (87.5)	1 (12.5)		8
	Clear cell carcinoma	5 (55.6)	4 (44.4)		9
	Mesonephric-like carcinoma	0 (0)	1 (100)		1
Vulva					3
	Squamous Cell Carcinoma	3 (100)	0 (0)		3

**Fig. 2:**
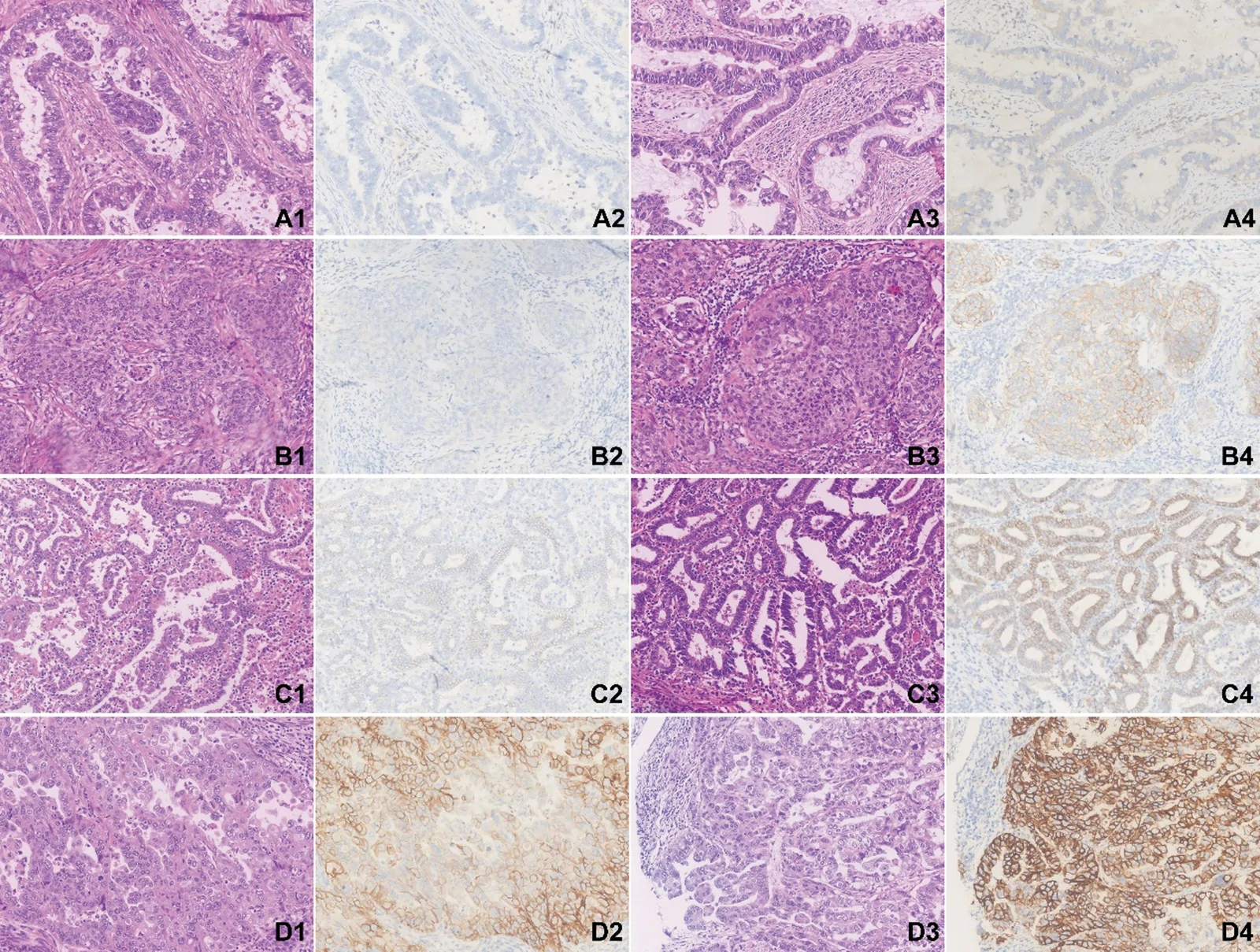
Representative intratumoral HER2 IHC discordance between dual/multiple tumor blocks. (**A**) Case showing HER2 score transition from 0 (Block 1: A1, H&E; A2, IHC) to 1+ (Block 2: A3, H&E; A4, IHC). (**B**) Case with HER2 score change from 0 (B1, H&E; B2, IHC) to 2+ (B3, H&E; B4, IHC). (**C**) Case demonstrating a shift from 1+ (C1, H&E; C2, IHC) to 2+ (C3, H&E; C4, IHC). (**D**) Case showing a progressive increase from 2+ (D1, H&E; D2, IHC) to 3+ (D3, H&E; D4, IHC)

In discordant cases, HER2 scores typically exhibited a stepwise increase (Table 2; Fig. 1F). Uterine tumors most commonly shifted from IHC 0 to 1+ (*n* = 13), 1 + to 2+ (*n* = 6), and 2 + to 3+ (*n* = 2). Similar transitions were observed in cervical carcinomas: 17 cases shifted from 0 to 1+, 2 from 0 to 2+, and 7 from 1 + to 2+. Ovarian tumors demonstrated shifted from 0 to 1+ (*n* = 22), 0 to 2+ (*n* = 2), 1 + to 2+ (*n* = 14), and 1 + to 3+ (*n* = 1). Representative figures of discordant HER2 expression are illustrated in Fig. 2.

**Table 2:** HER2 expression transitions from different blocks in gynecological tumor sites

Site	Block 1	Block 2	Cases amounts
Uterus			21
	0	1	13
	1	2	6
	2	3	2
Cervix			26
	0	1	17
	0	2	2
	1	2	7
Ovary			39
	0	1	22
	0	2	2
	1	2	14
	1	3	1

Dual-block assessment improved HER2 expression detection compared with single-block evaluation (Table 3). For HER2 3 + cases, dual-block assessment identified 1.0% of tumors, compared with 0.5% in Block 1 and 0.7% in Block 2; however, these differences were not statistically significant (Block 1 vs. dual-block: *P* = 0.571; Block 2 vs. dual-block: *P* = 1.000). In contrast, dual-block assessment significantly increased the detection of HER2 2 + expression to 15.6%, compared with 13.7% in Block 1 and 10.6% in Block 2. Compared with dual-block assessment, single-block evaluation showed significantly lower HER2 2 + detection rates (Block 1 vs. dual-block: *P* = 0.034, rate difference: 1.92%, 95% CI: 0.44% to 3.40%; Block 2 vs. dual-block: *P* < 0.001, rate difference: 5.05%, 95% CI: 2.84% to 7.25%). Similarly, the proportion of HER2 1 + cases increased from 30.5% in Block 1 and 31.5% in Block 2 to 33.9% with dual-block assessment. Compared with dual-block evaluation, Block 1 showed a significantly lower HER2 1 + detection rate (*P* = 0.034, rate difference: 3.37%, 95% CI: 0.72% to 6.01%), whereas the difference between Block 2 and dual-block assessment was not statistically significant (*P* = 0.259, rate difference: 2.40%, 95% CI: −0.85% to 5.66%). Conversely, the proportion of HER2 0 cases decreased from 55.3% in Block 1 and 57.2% in Block 2 to 49.5% with dual-block assessment, indicating a significant reduction in HER2-negative classification (Block 1 vs. dual-block: *P* < 0.001, rate difference: −5.77%, 95% CI: −8.01% to − 3.53%; Block 2 vs. dual-block: *P* < 0.001, rate difference: −7.69%, 95% CI: −10.25% to − 5.13%). Expanding HER2 testing to include dual-block analysis revealed that up to 13.5% of patients initially classified as HER2-0 by single-block evaluation exhibited detectable HER2 expression upon additional block assessment. Specifically, HER2 1+, 2+, and 3 + expression increased by 5.8%, 6.9%, and 0.8%, respectively. Importantly, many reclassified cases were HER2 1+, which may not necessarily confer eligibility for treatment with all HER2-targeted ADCs, as therapeutic benefit may vary according to HER2 expression level and specific ADC indications in gynecologic malignancies [13, 14].

**Table 3:** Comparison of single block and dual block assessment in total patients

HER2 status	Block1	Block2	Dual-block	*P* value (Block1 vs. Dual-block)	Rate difference %_(95% CI)	*P* value (Block2 vs. Dual-block)	Rate difference %_ (95% CI)
HER2 3+, n (%)	2 (0.5)	3 (0.7)	4 (1.0)	*P* = 0.571	0.48% (-0.18% to 1.15%)	*P* = 1.000	*0.24% (-0.23% to 0.71%)*
HER2 2+, n (%)	57 (13.7)	44 (10.6)	65 (15.6)	*P* = 0.034	*1.92% (0.44% to 3.40%)*	*P* < 0.001	*5.05% (2.84% to 7.25%)*
HER2 1+, n (%)	127 (30.5)	131 (31.5)	141 (33.9)	*P* = 0.034	*3.37% (0.72% to 6.01%)*	*P* = 0.259	*2.40% (-0.85% to 5.66%)*
HER2 0, n (%)	230 (55.3)	238 (57.2)	206 (49.5)	*P* < 0.001	*-5.77% (-8.01% to -3.53%)*	*P* < 0.001	*-7.69% (-10.25% to -5.13%)*

## Discussion

HER2, encoded by the ERBB2 gene, is a transmembrane tyrosine kinase receptor. Its overexpression, with or without gene amplification, drives aberrant signaling, cellular transformation, and tumorigenesis [15]. In gynecologic malignancies, HER2 serves as both a prognostic biomarker and a therapeutic target, guiding informing eligibility for trastuzumab-based treatments when HER2 overexpression or HER2 amplification [16]. Encouraging results from the phase II, open-label, multicenter DESTINY-PanTumor02 (DP-02) trial have led the National Comprehensive Cancer Network (NCCN) now recommends trastuzumab deruxtecan (T-DXd) as a therapeutic option for patients with recurrent ovarian, endometrial, and cervical cancers with HER2 expression [4]. Therefore, accurate assessment of HER2 status is critical for optimal patient selection.

HER2 expression exhibits considerable variation across different gynecologic histotypes and anatomical sites [17]. Low HER2 expression is commonly observed in gynecological malignancies [3]. In cervical adenocarcinomas, HER2 overexpression (IHC 3+) is observed in approximately 11% of cases, with gene amplification present in around 6%, rising to 14.7% in gastric-type adenocarcinomas [18]. Among the 22 cervical carcinoma cases analyzed in our cohort, no HER2 3 + expression was detected, and only two cases were classified as gastric-type adenocarcinoma. Endometrial serous carcinomas exhibit the highest rates of HER2 overexpression (38%) and amplification (8%) [19]. Similarly, recurrent or metastatic endometrial cancers demonstrate elevated HER2 positivity (35.6%) and amplification (15.6%) [20]. In ovarian cancer, HER2 overexpression or amplification is most prevalent in mucinous carcinoma, reaching up to 18.8% to 25% [21, 22], and with 26% intratumoral expression heterogeneity [21], while lower rates are observed in endometrioid carcinoma (2%) and clear cell carcinomas (4%~5.7%) [23–25]. In our cohort, HER2 overexpression (3+) was most frequently identified in uterine serous carcinoma (0.5%, 2/416), endometrioid carcinoma (0.2%, 1/416), and ovarian clear cell carcinoma (0.2%, 1/416). HER2 positivity has been consistently linked to poor prognosis across multiple gynecologic malignancies. In uterine serous carcinoma, HER2 overexpression serves as an independent prognostic marker, associated with higher recurrence rates and worse progression-free and overall survival [26]. Similarly, high HER2 expression in ovarian cancer is associated with advanced stage, lymph node metastasis, and inferior outcomes [27, 28]. In cervical adenocarcinoma, HER2 amplification is linked to advanced FIGO stage, perineural invasion, and ovarian metastasis, while IHC 3 + is more frequent in tumors with lymphovascular invasion [18]. In gastric-type cervical adenocarcinoma, HER2 positivity predicts shorter progression-free survival, and in cervical squamous cell carcinoma, it correlates with advanced stage, high grade, and nodal involvement [29, 30]. Notably, even low or negative HER2 expression holds prognostic value; in stage I/II uterine carcinosarcoma, HER2-low (defined as IHC 1+) and HER2-negative status (IHC 0) independently predict poorer survival [31]. These findings reinforce the prognostic relevance of HER2 across gynecologic malignancies and support its role as a therapeutic target.

HER2 heterogeneity has been well characterized in breast and gastric cancers, where intratumoral and intertumoral variability critically influences therapeutic efficacy [11]. Several studies have reported HER2 genetic heterogeneity in breast cancer, with frequencies ranging from 1% to 34% [32]. A retrospective analysis of 618 gastric cancer patients revealed intratumoral HER2 heterogeneity in 50% of tumors and intertumoral heterogeneity in 30% [6]. However, despite increasing recognition of HER2 heterogeneity in other malignancies, its systematic evaluation in gynecologic cancers remains limited. In this study, we conducted the first systematic assessment of intratumoral HER2 heterogeneity using dual paraffin blocks from 416 gynecologic tumors, evaluated by immunohistochemistry. HER2 expression discordance was observed in 20.7% cases, highlighting substantial spatial heterogeneity. Discordance rates varied by anatomical sites: uterine (23.9%), ovarian (21.4%), and cervical (18.2%) cancers. Histological subtype-based stratification revealed marked intertumoral discrepancies in HER2 status assessment, particularly highlighting intratumoral heterogeneity within specific cancer subtypes. HER2 expression between primary and recurrent/metastatic lesions also showed notable variability, with 43% of cases demonstrating discordant in endometrial cancer [33]. Similar findings were reported in uterine serous carcinoma, which showed marked HER2 intratumoral heterogeneity, a high IHC/fluorescence in situ hybridization (FISH) discordance rate, and divergent HER2 status between serous endometrial intraepithelial carcinoma (SEIC) and its invasive counterpart [34].

HER2 immunohistochemical testing, widely applied in breast and gastric cancers, commonly employs antibody platforms such as HercepTest™ (Dako) and PATHWAY^®^ HER2/neu 4B5 (Ventana). However, considerable variability in HER2 assessment persists across tumor types, potentially reflecting differences in antibody performance, scoring systems, detection platforms, and underlying biological heterogeneity. In breast cancer, the HercepTest monoclonal antibody has demonstrated greater sensitivity in detecting HER2 expression in tumors with gene amplification (ISH group 1), increased HER2 copy number (ISH group 4), and HER2-low disease (IHC 1 + or IHC 2+/ISH-negative) [35, 36]. By contrast, in gastric cancer, the Ventana 4B5 rabbit monoclonal antibody has shown superior sensitivity (95.2%) compared with SP3 and Dako HercepTest, whereas limited sampling approaches, such as tissue microarrays, may substantially underestimate HER2 positivity because of focal staining patterns [37]. In gynecologic malignancies, particularly endometrial and ovarian clear cell carcinomas, marked heterogeneity has been reported among HER2 antibody clones, with substantial discordance between IHC results and HER2 gene amplification assessed by in situ hybridization [38]. These findings suggest that HER2 assessment in gynecologic tumors may be particularly susceptible to both analytical and biological variability. Given the clone-specific differences in sensitivity for detecting weak or heterogeneous HER2 expression, the discordance observed between tissue blocks in our study may, at least in part, reflect assay-dependent variability in addition to intrinsic tumor heterogeneity. Further studies are therefore warranted to identify the most appropriate antibody platforms and optimize HER2 testing strategies in gynecologic malignancies. Tumor biomarker expression is inherently dynamic and heterogeneous, influenced by inter-patient genetic variability, molecular regulatory mechanisms, and tumor microenvironmental modulation. Previous studies across different malignancies have highlighted that biomarker expression and prognostic evaluation may be substantially affected by biological variability and sampling-dependent heterogeneity, thereby influencing therapeutic response and clinical interpretation [39, 40]. Furthermore, dynamic changes in gene expression may contribute to variability in protein-level detection and biomarker classification [41]. Tumor sensitization and treatment responsiveness are also shaped by microenvironmental signaling networks, including cytokine-mediated interactions [42], while inflammatory signaling pathways may further modulate tumor biology and molecular expression patterns [43]. Collectively, these findings emphasize the biological complexity of HER2 expression and the need for standardized testing protocols in gynecologic cancers.

The recognition of HER2 heterogeneity, coupled with the evolving concept of ultra-low HER2 expression, challenges the traditional binary classification. Dual-block assessment represents an effective and efficient strategy to enhance HER2 detection sensitivity. However, the ultimate goal is not merely to increase positivity rates but to identify patients likely to benefit from HER2-targeted therapies. Emerging evidence suggests that patients with HER2-low or even ultra-low expression may still derive clinical benefit from HER2-targeted ADCs [44]. In our series, expanding HER2 assessment to a dual-block strategy reclassified up to 13.5% of cases initially categorized as HER2-0 by single-block evaluation into HER2-expressing tumors, potentially enlarging the population eligible for HER2-targeted therapies. Notably, this increase was primarily driven by gains in HER2 1+ (5.8%) and HER2 2+ (6.9%) expression, with an additional 0.8% increase in HER2 3 + cases, further underscoring the clinical relevance of enhanced sampling for capturing spatial heterogeneity. Dual-block HER2 testing may enhance detection sensitivity by capturing spatial heterogeneity. Previous studies have demonstrated that placing two tissue blocks on a single slide allows simultaneous IHC evaluation without increasing reagent consumption [6, 10]. In gynecologic oncology, where HER2-targeted therapies are gaining traction, incorporating strategies to assess spatial heterogeneity—such as dual-block IHC—may refine biomarker-driven treatment decisions and expand therapeutic opportunities.

Several limitations of this study warrant consideration. First, the retrospective design introduces inherent selection and information biases, as case inclusion depended on the availability of adequate archival FFPE material. Moreover, because HER2-targeted ADCs have not yet been widely implemented in routine gynecologic oncology practice, large-scale real-world cohorts evaluating the relationship between HER2 intratumoral heterogeneity and therapeutic response remain lacking. Consequently, our study was unable to assess correlations between HER2 heterogeneity and treatment outcomes. Prospective studies incorporating standardized treatment regimens and longitudinal follow-up will be necessary to establish the therapeutic relevance of HER2 spatial heterogeneity. Second, although FISH remains the gold standard for confirming HER2 gene amplification, particularly in equivocal (IHC 2+) cases [45, 46], it was not systematically performed across the entire cohort, representing a methodological limitation. Nevertheless, the therapeutic landscape of HER2-directed oncology has evolved, with contemporary HER2-targeted therapies—particularly ADCs such as trastuzumab deruxtecan (T-DXd)—increasingly guided by HER2 protein expression rather than strict amplification status. Clinical evidence suggests that ADC efficacy may extend to HER2-low or IHC-defined populations [47–49]. Emerging evidence further suggests that HER2-targeted ADCs, including disitamab vedotin (RC48), may demonstrate activity in HER2-low gynecologic tumors (IHC 1+/2+) independent of amplification status [50, 51]. Therefore, despite the absence of comprehensive FISH validation, our IHC-based findings remain clinically informative within the evolving treatment paradigm of HER2-targeted therapy. Third, this analysis represents a single-center experience, and institutional variability in tissue handling, fixation, immunohistochemical protocols and antibody performance may limit the generalizability of the findings despite the use of standardized scoring criteria. Multicenter validation across diverse clinical settings is therefore required to confirm the robustness of the observed intratumoral heterogeneity patterns. Fourth, the distribution of gynecologic tumor subtypes within the cohort was imbalanced. While common tumor types were well represented, rare malignancies—such as vulvar cancer or mesonephric-like carcinoma—were included in very small numbers, rendering subgroup-specific conclusions statistically underpowered and necessitating cautious interpretation. Finally, HER2 immunohistochemistry is inherently semi-quantitative and subject to interobserver variability, particularly in low-expression categories. The nuanced distinction between HER2-0 and HER2-low (1+) remains susceptible to interpretation bias, highlighting the need for future integration of AI-assisted digital quantification. Although dual-block sampling improves spatial representation compared with single-block assessment, it still captures only a fraction of the tumor landscape, and additional heterogeneity may remain unsampled. Prospective multicenter studies bridging spatial heterogeneity with clinical outcomes are warranted to translate these findings into clinically actionable strategies for precision oncology.

In conclusion, our findings highlight the clinical relevance of HER2 intratumoral heterogeneity in surgically resected gynecologic malignancies, emphasizing the need for optimized pathological assessment in the postoperative setting. Incorporating enhanced HER2 testing strategies—such as dual-block IHC evaluation—may improve diagnostic precision and facilitate more accurate selection of candidates for HER2-targeted therapies. Further multidisciplinary studies are warranted to establish standardized HER2 assessment protocols and to elucidate the biological and therapeutic implications of HER2 heterogeneity for surgical oncology practice. 

## Supplementary Information

Below is the link to the electronic supplementary material.

Supplementary Material 1

## Data Availability

The datasets generated during and/or analysed during the current study are available from the corresponding author on reasonable request.
